# Around the Hospitals

**Published:** 1894-09-01

**Authors:** 


### Around the Hospitals.

[Notice to the Managers of Institutions.—Special space is now reserved for the insertion of notices of hospital meetings and festivals. The Editor requests that all notices may reach The Hospital Office, 428, Strand, at least one week before the date of each meeting.]

**Chester General Infirmary.**—The report of the Chester Royal Infirmary for 1893 is an especially interesting one, and may be remarked for the spirit of appreciation which shows itself in its pages. It must be satisfactory to the officers of the institution, honorary and otherwise, to feel and realise that their services should be recognised and valued as they evidently are. A good many useful and necessary improvements have been carried out during the past year, first in magnitude and importance being the completion of the new drainage and sanitary systems, which it is now hoped have been brought to "the latest standard of approved sanitary regulations." The difficulties of putting this work through have been great, and the cost has been somewhat higher than was at first estimated, but the board rightly considered that "no sacrifice was too great to make in order to remedy the serious defects discovered." To enable these alterations to be made the sum of £2,038 18s. 7d. Consols has been sold out at 98¼, realizing the sum of £2,000. The annual income from invested funds has thus been necessarily reduced, but it is satisfactory to note an increase of £20 in the annual subscriptions over last year. The private nursing department is doing well, a credit balance appearing in favour of the charity from this source of £206 2s. 1d., after deducting all expenses of wages, uniform, &c. The average daily number of occupied beds throughout the year was in excess of the previous one, 115 as against 94, while the average cost per occupied bed is less, being £45 15s. 7d., as against £48 15s. 3d. in the previous year. Among improvements may be mentioned two new balconies in front of the infirmary building, which will be a source of great pleasure to the patients, and better the ventilation of the adjoining wards. The money for these came through Captain Mascie Taylor, being a portion of a sum left to him for charitable purposes by the late Miss Sara Hester Jones. The reflooring and renovating of one of the wards has been carried out by the kindness of the officers of the Earl of Chester's Yeomanry Cavalry, in memory of a brother officer. The Convalescent Home at Park Gate is an important feature of this hospital, and is doing a good work. Under the superintendence of Miss Barrow the nursing staff maintain an efficiency which entitles them to the committee's grateful thanks, which are also extended to the medical staff and to many kind friends.

